# Assessing needs for psychiatric treatment in prisoners: 1. Prevalence of disorder

**DOI:** 10.1007/s00127-016-1311-7

**Published:** 2016-11-22

**Authors:** Paul Bebbington, Sharon Jakobowitz, Nigel McKenzie, Helen Killaspy, Rachel Iveson, Gary Duffield, Mark Kerr

**Affiliations:** 1 UCL Division of Psychiatry, Faculty of Brain Sciences, University College London, 6th Floor Maple House, 149 Tottenham Court Road, London, W1T 7NF UK; 2 Camden and Islington Foundation Trust, St Pancras Hospital, 4 St Pancras Way, London, NW1 OPE UK; 3 North London Forensic Service, Chase Farm Hospital, The Ridgeway, Enfield, Middlesex EN2 8JL UK; 4 Faculty of Social Sciences, School of Social Policy, Sociology and Social Research, Cornwallis North East, University of Kent, Canterbury, Kent CT2 7NF UK

**Keywords:** Prisoners, Epidemiology, Prevalence, Psychosis, Psychiatric disorders, Substance abuse, Needs for care

## Abstract

**Background:**

High levels of psychiatric morbidity in prisoners have important implications for services. Assessing Needs for Psychiatric Treatment in Prisoners is an evaluation of representative samples of prisoners in a male and a female prison in London. This paper reports on the prevalence of mental disorders. In a companion paper, we describe how this translates into mental health treatment needs and the extent to which they have been met.

**Methods:**

Prisoners were randomly sampled in a sequential procedure based on the Local Inmate Data System. We interviewed roughly equal numbers from the following groups: male remand; male sentenced prisoners (Pentonville prison); and female remand; female sentenced prisoners (Holloway prison). Structured assessments were made of psychosis, common mental disorders, PTSD, personality disorder and substance abuse.

**Results:**

We interviewed 197 male and 171 female prisoners. Psychiatric morbidity in male and female, sentenced and remand prisoners far exceeded in prevalence and severity than in equivalent general population surveys. In particular, 12% met criteria for psychosis; 53.8% for depressive disorders; 26.8% for anxiety disorders; 33.1% were dependent on alcohol and 57.1% on illegal drugs; 34.2% had some form of personality disorder; and 69.1% had two disorders or more. Moreover, in the year before imprisonment, 25.3% had used mental health services.

**Conclusions:**

These rates of mental ill-health and their similarity in remand and sentenced prisoners indicate that diversion of people with mental health problems from the prison arm of the criminal justice system remains inadequate, with serious consequences for well-being and recidivism.

## Introduction

The boundary between prison and the outside community appears to be particularly permeable to people with mental health problems, whose behaviour sometimes leads them to the attention of the criminal justice system. The consequential increased prevalence of mental disorder in prisoners was identified in a number of early studies [1–5]. The British National Survey of Psychiatric Morbidity in Prisons (NPMS-P [6]) applied the relatively sophisticated methods of the National Household Survey of Psychiatric Morbidity [7] to a sample drawn from every prison in England and Wales, thereby allowing direct comparisons with the general population [8–12]. It confirmed that levels of psychiatric morbidity, ranging from common mental disorders to psychosis, personality disorder and drug and alcohol problems, were greatly in excess in prisoners. At around 10%, the prevalence of psychosis was particularly troubling, and clearly demands further investigation. In particular, it is at variance with the results of systematic reviews of psychiatric morbidity in prisoners [13].

This morbidity is associated with increased rates of victimization, both violent and sexual, and has a major impact on the prisoners in terms of suicidal ideation and behaviour and deliberate self-harm [14]. It often occurs in the context of serious social exclusion. It has long been argued that the way prisons are run is detrimental to the rehabilitation of prisoners with serious mental health problems, and that they must receive adequate medical care, both on grounds of humanity and with a view to reducing recidivism. Indeed, imprisonment is plainly inappropriate for many offenders with mental disorders and, where possible, diversion at various stages of the justice process is accepted as more productive [15].

While prison mental health services in the UK did improve after the millennium, investment in such services should be guided by a clear account of the actual treatment needs of prisoners and their overall scale. The Bradley review [15] (2009) called for a repeat of NPMS-P to provide up-to-date data for treatment provision strategies. The government accepted this recommendation, but no such study has yet been commissioned. The current study (Assessing Needs for Psychiatric Treatment in Prisoners; ANPTP) aimed to address this gap by providing at least local data to compare with the findings of NPMS-P, in particular those relating to psychosis. Our further purposes were to quantify the corresponding need for mental health care and treatment in male and female prisoners, and to assess the extent to which this was met by the various mental health facilities in prison [16].

ANPTP was carried out in two London prisons dealing with locally remanded and sentenced prisoners, in which the responsibility for psychiatric services lay with local NHS trusts. Holloway then had an operational capacity of 512, while HMP Pentonville accommodated around 1200 male prisoners. Psychiatric services in these prisons were well organised at the time of the project, as described more fully in the companion publication [16]. In this initial paper, we describe the general methods involved in the study, and report the frequency of psychiatric morbidity by sex and sentencing status. If there is, indeed, effective pre-sentence diversion of people with mental disorders, we would predict, specifically, that levels of psychiatric disorder will be higher in remand than in sentenced prisoners.

## Methods

### Sample

The study involved the establishment of a sample of inmates in each prison, interviewed in a single phase. Prisoners were selected from the following groups, which we attempted to sample in equal numbers: male remand; female remand; male sentenced; female sentenced.

There are different ways in which samples of prisoners might be established. Prison stays vary in length and are bracketed by the events of incarceration and release. Establishing a sample at a single point in time to last for the whole duration of the assessment period will, thus, result in progressive attrition of the sample, with an increasing failure rate. Failure will not be random, as there will be selective loss of inmates with short sentences, who may have higher rates of psychiatric disorder. Such a sampling procedure therefore provides an estimated prevalence skewed towards inmates with long sentences. This estimate may be of some use, as it reflects the routine day-to-day level of work required from medical teams providing care. However, the work of care is increased at the points of incarceration and release, so this approach may underestimate resource requirements. Our chosen strategy was therefore to use sequential sampling, refreshing the sampling frame every 2 weeks. This maximized both our capture of short-stay inmates and our use of research resources. The resulting prevalence rates are therefore likely to be higher than those using the single-sample approach, but will also be a more accurate representation of the resources required to provide care.

Sampling was based on the Local Inmate Data System (LIDS, the computerized register of all prisoners). We used fortnightly census points: a sample of five sentenced and five remand prisoners was chosen at random, with the intention of seeing them within the subsequent 2 weeks. The selected prisoners were approached, given an information leaflet about the research, and invited to give their informed written consent to participate. Prisoners who had been moved, were otherwise unavailable within a foreseeable period, or refused to participate, were replaced by a subsequent individual selected at random to ensure the required numbers for each 2-week period. Consistent with the high turnover, no prisoner was sampled twice.

On average, interviews took about 2 h to complete, and were sometimes conducted in more than one session. They were carried out by SJ (a psychologist) between September 2007 and December 2009. She received extensive training in the administration of SCAN and SCID-II from PB and NM. This included watching video assessments and the completion of mock assessments for review by the trainers.

### Instruments

The Revised Version of the Clinical Interview Schedule (CIS-R [17]) assesses neurotic symptoms and common mental disorders in the week preceding interview. It defines six neurotic disorders: depressive episode; generalised anxiety disorder; mixed anxiety and depressive disorder; phobia; panic disorder; and obsessive–compulsive disorder. Diagnosis is established by applying algorithms based on the ICD-10 diagnostic criteria for research [18]. The instrument took on average about 25 min to administer.

ICD-10 diagnoses of psychotic disorders were determined using relevant sections of the SCAN interview [19, 20], i.e. those covering expansive mood and ideation; hallucinations; subjectively described disorder of thought and experiences of replacement of will; and delusions. The interview covered the 1-year period before interview. The instrument incorporates a short screening section that takes about 5 min to complete. Participants were usually screened out, but if not, the full instrument took 20–60 min.

Personality disorder was assessed as in NPMS-P, that is, with the Structured Clinical Interview for DSM-IV (SCID-II [21]). This is based on the DSM-IV Axis II classification system [22]: the rationale for not using the ICD-10 classifications is that set out in NPMS-P report (p. 33) [6]. However, NPMS-P was based on a two-stage procedure, with lay interviewers initially using the self-report screening version; clinicians then administered the full version of the SCID to a 1-in-5 sub-sample. In the current survey, we used the full version with all participants in order to maximize validity.

The SCID-II clinical interview covers each personality disorder category in turn and, within each category, each component criterion is evaluated by a specified probe (or probes) and subsequent specified questions. It has 120 items rated on a four-point scale: ‘inadequate information’, ‘negative’, ‘sub-threshold’, and ‘threshold’. There are 12 modules (plus a ‘not otherwise specified’ category), covering avoidant, dependent, obsessive–compulsive, paranoid, schizotypal, schizoid, histrionic, narcissistic, borderline, antisocial, passive–aggressive, and depressive personality disorders. As in NPMS-P, we omitted depressive and passive–aggressive personality disorders, as they are not included in DSM-IV. The instrument typically took around 25 min to complete.

Post-traumatic stress disorder (PTSD) was assessed with the Posttraumatic Stress Diagnostic Scale Manual (PDS [23]). This self-report measure contains 49 items. A short checklist identifies potentially traumatizing events experienced by the respondent. If they feel that they have been affected by such an event in the preceding month, symptoms characteristic of PTSD are assessed in terms of their onset and severity, and of their impact on skills of daily living. This information is used to make a diagnosis based on DSM criteria, i.e. a person must meet one or more diagnostic criteria within each of the six required sets.

Alcohol misuse and dependence: As in the Adult Psychiatric Morbidity Surveys [24], we used the Alcohol Use Disorders Identification Test (AUDIT [25]) to assess hazardous and harmful misuse of alcohol, taking the year before imprisonment as the reference period. The ten questions in the AUDIT are scored from zero to four and summed to provide a total score. A score of eight indicates hazardous alcohol use.

However, alcohol dependence was assessed using the Severity of Alcohol Dependence questionnaire (SAD-Q [26]). Twenty questions cover a range of symptoms of dependence. Scores range from 0 to 3 on each question. A score of 4–19 indicates mild dependence, 20–34 moderate dependence, and 35–60 severe dependence. The reference period was again the year before imprisonment. Both the AUDIT and the SAD-Q rely on self-completion by the respondents, and each took about 10 min.

Drug dependence: We used the questions developed to measure drug use in the Adult Psychiatric Morbidity Surveys [24]. Information was collected on respondents’ drug use in the year before imprisonment, covering cannabis, amphetamines, crack, cocaine, ecstasy, tranquillizers, opiates and volatile substances, such as glue. Five questions measure dependence on each individual drug. A positive response to any of the five questions was taken to indicate drug dependence.

In the current report, we present straightforward comparisons of the prevalence of psychiatric disorders: discrepancies between the sexes may indicate whether mental disorders in offenders are handled differently in the criminal justice system, while those between remand and sentenced prisoners speak to the effectiveness of attempts to divert disordered offenders from the process of incarceration.

## Results

### Response rates

Approximately ten sentenced and ten remand prisoners were sampled per month, of whom 5–8 would complete the whole assessment process. The overall response rate was 70.0%. Most failures were due to unpredicted unavailability, rather than to refusal. The final sample size was 368 (197 males, 171 females). Our intention to sample equal numbers of remand and sentenced prisoners was achieved: 46.7% of male prisoners and 48.0% of females were on remand.

### Socio-demographic characteristics

Of male study participants, 1.0% were aged from 18 to 20, 45.9% from 21 to 30, and 53.1% over 30. The corresponding figures for females were 17.0, 36.2 and 46.2%. There were more offenders aged 18–20 in the Holloway sample, as it incorporated a Young Offender Institution, whereas Pentonville did not. Approximately, a third of the prisoners in the current sample were black or black/white mixed, closely comparable with London prisons as a whole (32%).

### Prior psychiatric service use

Psychiatric service usage in our sample was strikingly high in the year before imprisonment (see Table 1). A quarter had been in touch with mental health services, 7.4% reported a period of admission to a psychiatric hospital, and 38% had a keyworker (not necessarily provided by mental health services). While men had twice the psychiatric admission rates of women, the difference was non-significant. However, significantly more women had a keyworker in the community (*p* < 0.01). Rates of contact with mental health services and hospital admission were similar in sentenced and remand prisoners; however, significantly more sentenced prisoners reported having a keyworker.

**Table 1:** Rates of mental health service use in the 12 months prior to imprisonment

Service	Contact with mental health services	Psychiatric hospital admission	Keyworker
Men	22.3% (44/197)	9.2% (18/196)	31.1% (61/196)^††^
Women	28.7% (49/171)	5.3% (9/171)	45.6% (78/171)^††^
Sentenced	23.2% (45/194)	6.2% (12/194)	43.3% (84/194)^†^
Remand	27.6% (48/174)	8.7% (15/173)	31.8% (55/173)^†^
Total	25.3% (93/368)	7.4% (27/367)	37.9% (139/367)

^†^Female prisoners significantly more than male prisoners (*p* < 0.01)

^††^Sentenced significantly more than remand (*p* < 0.05)

### Current psychiatric morbidity

Table 2 shows individual diagnoses, broken down by gender and sentencing status. Gender differences were less marked than might have been expected: morbidity rates were consistent across the sexes, except for PTSD and phobias, which were more than twice as frequent in women (*p* < 0.01 in both cases). The overall difference between remanded and sentenced prisoners was also small.

**Table 2:** Proportion of respondents who met diagnostic criteria

Disorder	Men	Women	Sentenced	Remand	Total
Psychosis	14.2% (28/197)	9.9% (17/171)	8.8% (17/194)*	16.1% (28/174)*	12.2% (45/368)
Depressive states^†^	49.2% (97/197)	58.0% (98/169)	50.0% (97/194)	57% (98/172)	53.8% (197/366)
Depressive episode	20.3% (40/197)	24.6%(42/171)	26.3% (51/194)	17.8% (31/174)	22.3% (82/368)
Anxiety states^††^	29.1% (57/196)	24.3% (41/169)	28.4% (55/194)	25.1 (43/171)	26.8% (98/365)
Phobias	6.6% (13/197)**	16.0% (27/169)**	11.9% (23/194)	9.9% (17/172)	10.9% (40/366)
Panic	5.1% (10/197)	5.9% (10/169)	4.6% (9/194)	6.4% (11/172)	5.5% (20/366)
PTSD	4.6% (9/197)**	12.0% (20/166)**	7.3% (14/191)	8.7% (15/172)	8.0% (29/363)
Personality disorder	35.5% (70/197)	32.7% (56/171)	29.9% (58/194)	39.1% (68/174)	34.2% (126/368)
Alcohol dependence	32.0% (63/197)	34.3% (58/169)	33.5% (65/194)	32.6% (56/172)	33.1% (121/366)
Drug Dependency	54.8% (108/197)	59.6% (102/171)	54.1% (105/194)	60.3% (105/174)	57.1% (210/368)

^†^Depressive states: depressive episode plus mixed anxiety/depressive disorder

^††^Anxiety states: generalised anxiety disorder, phobia, panic

Indicated pairs * *p* < 0.05; ** *p* < 0.01

The overall prevalence of psychosis was extremely high, higher in men than in women (14.2 versus 9.9%) though not significantly so. The frequency of psychosis in remand prisoners was nearly twice that in their sentenced counterparts; even so, the rate in the latter was still around 9% (NS).

Depressive states as a whole (depressive episode plus mixed anxiety/depression) were somewhat higher in female than in male prisoners, but remarkably high in both, at approximately 50%. The prevalence of the more severe form, depressive episode, is particularly striking, affecting over 20% of prisoners. The prevalence of depressive states was slightly greater in remand than in sentenced prisoners. Anxiety disorders (defined as generalised anxiety disorder, phobia, or panic) were also extremely common in both men and women, approaching 30%. There was no sex difference in the prevalence of panic attacks, but phobias were more than twice as frequent in women (*p* < 0.01), as was PTSD (*p* < 0.01). There were no significant differences between remand and sentenced prisoners in the rates of these various affective conditions.

Around one-third of prisoners of each sex and in each sentencing category met criteria for at least mild alcohol dependence in the year before incarceration. The prevalence of hazardous drinking was identical in men and women (52%). Drug dependence was very frequent indeed, in both sexes and both sentencing categories. It was identified in 55% of male and 60% of female prisoners.

As expected, personality disorders were common. Borderline and antisocial types were the most frequent, the former more so in women, the latter in men. Avoidant personality disorder was the only other relatively frequent category, particularly in women; it was slightly more common in sentenced than in remand prisoners. Paranoid personality disorder was identified in only 1% of men and 2% of women.

Overall, remand prisoners had a somewhat greater prevalence of personality disorders than their sentenced counterparts (particularly borderline and antisocial personality disorder), but not significantly so.

**Fig. 1:**
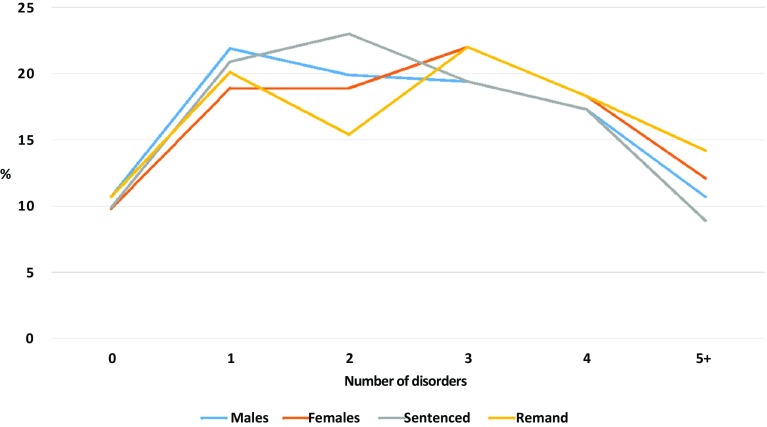
Psychiatric comorbidity in prisoners by sex and sentencing type

Figure 1 makes very clear the high frequency with which disorders co-occur in these prison populations. Only 10.3% of respondents did not meet diagnostic criteria for at least one disorder: 70% had two or more disorders and 11.7% met criteria for five or more disorders. Rates of comorbidity were similar in males and females, and in sentenced and remanded prisoners. Of depressed respondents, 61.5% were dependent on drugs and 59% were drinking hazardously before being imprisoned. This relationship was bi-directional, as 56.0% of prisoners reporting hazardous drinking or drug dependence were also classed as depressed. Personality disorder was similarly comorbid with drug dependence and drinking at hazardous levels.

## Discussion

### Characteristics of the sample

The catchment area for the prisons in this study is effectively that of the London courts served by them. It includes some of the most socio-economically deprived and ethnically diverse boroughs in the UK. As a result, the proportion of black and black/white mixed prisoners closely resembled that in other London prisons, but was more than twice that for prisons in England and Wales as a whole [27]. It greatly exceeds the proportion of black and black/white mixed groups in the general population: 4.4% in England and Wales, and 15.6% in the London area according to the 2011 census. The age distribution of prisoners was similar to those in NPMS-P [6], and in line with norms for the England and Wales prison population, which is predominantly young [28].

### Contact with psychiatric services

Participants had very high rates of recent pre-imprisonment contact with mental health services: a quarter had been in touch with mental health services and around 7% had been admitted to a psychiatric hospital. As the equivalent English population rates were 2.7 and 0.26% at the time of our survey [29], the prison values were ten and 42 times the population rate, respectively. Many participants had a keyworker in the community prior to their imprisonment, especially female and sentenced prisoners. Such contact had, by definition, been insufficient to keep them out of the criminal justice system.

### Prevalence of psychiatric disorder

This level of prior contact with services is commensurate with the very high prevalence of disorders identified in our sample. As indicated above, our sampling strategy is likely to result in prevalence rates somewhat higher than those using the single-sample approach. In Table 3, we provide comparisons with NPMS-P [6], and with the English Adult Psychiatric Morbidity Survey of 2007 [24], together with data from systematic reviews of international studies of mental disorder in prisons [13, 30, 31]. Overall, the gender differences in our survey were less marked than would be expected from general population rates. In fact, morbidity rates were generally consistent between the sexes, and the differences in this sample were not statistically significant (with the exception of PTSD and phobias). This suggests that the increased permeability of the prison/community boundary to people with mental health problems is greater in men.

**Table 3:** Comparison of ANPTP results with other relevant findings

Disorder	ANPTP (%)	NPMS-P (%)	Fazel & Seewald 2012 (%)	Fazel & Baillargeon 2011 (%)	Fazel & Danesh 2002 (%)	APMS 2007 (%)
Psychosis						
Men	14.2	8.4	3.6	4	4	0.3
Women	9.9	13.8	3.9	4	4	0.5
Depressive states*						
Men	49.2	35.5				8.8
Women	58.0	48.6				13.8
Depressive episode						
Men	19.9	12.8	10.2	10	10	1.9
Women	23.7	16.5	14.1	12	12	2.8
Anxiety states^†^						
Men	29.1	22.3				5.2
Women	24.3	27.9				8.5
Phobias						
Men	6.6	8.1				0.8
Women	16.0	12.7				2.0
Panic						
Men	5.1	4.6				1.0
Women	5.9	4.2				1.2
PTSD						
Men	4.6	4.1		4–21		2.6
Women	12.0	9.1		10–21		3.3
Antisocial personality disorder						
Men	26.4	55.2		65	47	0.6
Women	17.5	31.4		42	21	0.1
Borderline personality disorder						
Men	10.7	18.1				0.3
Women	15.2	20.0				0.6
Alcohol dependence						
Men	32.0	NA		18–30		8.7
Women	34.3			10–24		3.3
Drug dependence						
Men	54.8	47.2		10–48		4.5
Women	59.6	54.1		30–60		2.3

* Depressive episode plus mixed anxiety/depressive disorder

^†^Generalised anxiety disorder, phobia, panic

Both NPMS-P and the current study identified rates of psychiatric disorder much higher than in members of the community at large. This is particularly clear in relation to the more severe disorders: depressive episode and, notably, psychosis. This skew towards severity is apparent in the ratio between the prevalences of mixed anxiety/depression and depressive episode: while this is 3.7 in the general population, it is only 1.4 in our prison sample.

ANPTP and NPMS-P both found a very high prevalence of psychosis. This was over 20 times the 0.5% prevalence in the English population [32], and appears to have persisted for the 15 years separating the studies. However, in their systemic review, Fazel and Seewald [13] reported a much lower prevalence of 3.6% for psychosis. Some of this discrepancy may be the result of the selection criteria of the meta-analysis (the authors used a time-frame of 6 months before imprisonment for assessing psychosis, not the 12 months used here and in NPMS-P: thus the review excluded the 3000 prisoners with high rates of psychosis in NPMS-P). It is also possible that the international studies used less rigorous techniques of sampling and interviewing, or that, in some jurisdictions, there was something idiosyncratic about prisons suitable for accommodating psychiatric surveys. We should emphasize that psychosis in both NPMS-P and ANPTP was identified by trained interviewers using a standardised psychiatric interview (SCAN). Our results for depressive episode were also higher than those obtained in the various international systematic reviews carried out by Fazel and colleagues [13, 14, 30, 31], though the excess was less than for psychosis. Rates of depressive and anxiety disorders were similar in NPMS-P and the current study, though we found higher rates of PTSD in women.

High rates of psychiatric disorder might be expected in prisons like Holloway and Pentonville serving economically depressed inner city areas, as the social correlates of severe psychiatric disorder are very similar to those of criminal behaviour [33]. However, NPMS-P, with equally high rates, sampled from all prisons in England and Wales.

Figures for hazardous drinking are appreciably higher than the general population rate, but those for alcohol dependence are three times as high in males and ten times in females. In contrast to the general population, there was no sex difference. Thus, alcohol problems are also skewed towards the more severe end of the spectrum in prisoners, particularly in women. Drug dependence was equally widespread, and our figures stand in starkest contrast to the general population, representing a 12-fold increase in men and 26-fold in women [24].

Our overall prevalence rate for personality disorder was also high, with borderline personality disorder 33 times, and antisocial personality disorder 73 times more frequent than in the general population. Nevertheless, it was only half that in the NPMS-P sample [6]. However, this may be because the method of identifying personality disorders was different: we used the full version of the SCID-II, whereas the NPMS-P data were based only on the screening version, increasing the possibility of false positives. The discrepancy with the systemic reviews carried out by Fazel and colleagues in relation to antisocial personality disorder (Table 3) may also be due to methods of assessment [13, 31]. However, there was substantial heterogeneity between the included studies. This may originate from differences both in research methodology and in judicial policy.

Our data on comorbidity provide a final indicator of the burden of mental disorder in prisoners: in the general population, the sum total of disorders follows an exponential curve, with a majority having no disorders at all. Comorbidity was also very frequent in the meta-analysis of Fazel and Seewald [13].

It should be noted that the differences between sentenced and remand prisoners were small. Only psychosis and personality disorder were commoner in remand prisoners than in their sentenced counterparts, though the difference was only significant for psychosis. This may represent some, limited, success in diverting offenders with psychosis from custodial sentences.

However, the English prison population rose by 64% between 1993 and 2011 [34]. The rates of disorder in the current study are commensurate with those from NPMS-P more than 10 years previously. This implies a considerable absolute increase in the numbers of people with mental disorders in prison. It should be noted that the number of psychiatric beds declined by 44% over this period [34].

If our values for psychosis are valid, and in particular if the UK is indeed an outlier in international terms, it suggests an idiosyncrasy in the way the justice system in the UK deals with people with serious mental illness. It implies that current approaches to management are unjust and inefficient. In their latest paper, Fazel and colleagues [14] produce a comprehensive and plausible wish-list of suitable interventions in prisons. However, it is always difficult to deliver such treatments in the prison environment, and diversion is likely to be more efficient and just.

## Conclusions

Prisoners with mental health problems are in prison because they have been charged with or convicted of criminal acts. Crime in mentally ill people is driven by the same factors as in the mentally well, but magnified by the vulnerabilities caused by their illness [35]. Their mental condition renders them socially vulnerable, and as this vulnerability is persistent, they are prone to recidivism [35, 36]. Common humanity and the social imperative of reducing crime levels therefore converge on the need to offer effective treatments for prisoners with mental illness. The prison environment is not and never will be conducive to mental well-being, despite the commitment of medical, mental health and prison staff, apparent in our experience in these prisons. As far as possible, treatment should therefore be located outside prison, through diversion procedures. Probation services certainly deal with some offenders with mental health problems [37], but require more effective approaches [38]. There is also the hope that more effective community mental health services may reduce their clients’ exposure to the internal and external circumstances in which crime becomes more likely [39–41].

In the time since we carried out the ANPTP, the funding of prisons has been further reduced. The Home Office and Ministry of Justice budgets (which cover criminal justice in England and Wales) fell by 19 and 29%, respectively, between 2010 and 2014, paralleled by a reduction in overall UK spending on criminal justice by 12% [42]. This cannot but have a detrimental effect on the general suitability of the prison environment for managing prisoners with mental health problems, particularly as prisoner numbers remain high despite declining crime rates. It has been compounded by recent changes in the probation service in the UK [43].

Although it was a key recommendation of the Bradley report [15], NPMS-P has never been repeated. Our results strongly support the imperative for a new national survey, particularly in view of the doubling of the British prison population in the last fifteen years.
